# Heart Transplantation Following Fontan Failure: Long-Term Survival Analysis

**DOI:** 10.3390/jcm13102960

**Published:** 2024-05-17

**Authors:** Michele D’Alonzo, Federico Brunelli, Francesco Seddio, Francesca Julia Papesso, Rocco Davide Petruccelli, Roberta Di Cosola, Maurizio Merlo, Claudio Muneretto, Amedeo Terzi, Nicola Uricchio

**Affiliations:** 1 Cardiac Surgery Unit, Cardio-Thoracic Department, University of Brescia, 25123 Brescia, Italy; 2 Paediatric Cardiovascular Surgery Unit, Bergamo Hospital, 24127 Bergamo, Italy

**Keywords:** Fontan failure, heart transplantation, survival analysis, congenital heart disease, univentricular circulation

## Abstract

**Objectives:** Fontan circulation presents significant challenges for patients with congenital heart disease, often necessitating heart transplantation (HTX) due to deteriorating functionality across multiple organ systems. However, the impact of prior Fontan palliation on HTX outcomes remains poorly understood, with early mortality rates suggesting a heightened risk. The aim of our study is to evaluate the long-term results after heart transplantation in patients with univentricular congenital heart disease previously palliated with Fontan circulation. **Methods:** A retrospective analysis was conducted on patients who underwent HTX for congenital heart disease. Patients were categorized into two groups based on the pre-HTX circulation pathway: the Failing Fontan Group (FFG) and the Biventricular Congenital Group (BCG). Data were collected from patients between 1987 and 2018. Early and late outcomes, including survival rates, were assessed and critically analyzed. **Results:** Of the 66 patients, 29 (43%) had a failing Fontan palliation (FFG), and 37 had biventricular congenital diseases (BCG) before heart transplantation. Early mortality (30-day) was not statistically different between the two group. The overall survival rate was 82.6 ± 13.9% at 1 year, 79.0 ± 14.9% at 5 years, 67.2 ± 17.6% at 10 years and 63.2 ± 18.2 ± at 15 years for the FFG, and 86.1 ±11.4% at 1 year, 79.5 ± 13.7% at 5 years, 75.7 ± 14.9% at 10 years, 75.7 ± 14.9% at 15 years for the BCG, with no statistically significant difference (Mantel Cox *p* value: 0.69, 0.89, 0.52 and 0.39, respectively). Regarding Cox-regression analysis, the long-term survival rate was not affected either by previous Fontan surgery or by the era of heart transplantation (before vs. after the year 2000). **Conclusions:** Although heart transplantation after Fontan palliation showed a higher risk in the early post-operative period, the medium- and long-term survival rates are comparable with biventricular circulation patients. Despite the failing Fontan patients being a challenging set of candidates for transplantation, it is a reasonable option in their treatment.

## 1. Introduction

Patients born with univentricular congenital diseases can be successfully treated with a multistage surgical strategy which culminates in total cavo-pulmonary connection or Fontan circulation.

The Fontan procedure leads to a circulation pathway characterized by passive drainage of the systemic veins to the pulmonary circulation without the support of a sub-pulmonary ventricle: this arrangement allows for a reduction in both volume overload and cyanosis [1].

Since its first description [2], the Fontan procedure has undergone several refinements, leading to significant improvements in the survival of patients, with the 10-year overall survival being 94% for extracardiac conduit, 75% for lateral tunnel and 70% for atrio pulmonary connection [3]. However, in the setting of Fontan circulation, because of important circulatory changes, a series of end-organ complications are expected. It can be inferred that the Fontan procedure is primarily palliative. Elevated central venous pressure and impaired cardiac output are the hallmarks of cavo-pulmonary flow, which result in a cascade of pathophysiological consequences.

Exploring in depth, the absence of a sub-pulmonary ventricle results in greater central venous pressure, with the residual postcapillary kinetic energy (in the systemic venous system) driving transpulmonary blood flow. This results in a decreased and less efficient transpulmonary blood flow compared with a biventricular circulation [4]. Therefore, despite the improvement in the configuration of the Fontan circuit (from atrio-pulmonary connection to the Total Cavo pulmonary connection), alongside with the increased survival of univentricular patients [5], these patients live in a chronic heart failure state, which often culminates in a cardiovascular condition called “Fontan failure”.

The long-lasting systemic venous hypertension associated with persistent volume overload frequently leads to complications like chronic heart failure, protein-losing enteropathy, and acute decompensation [6], propelling the patients towards heart transplantation.

The spectrum of Fontan failure encompasses two distinct ends. One involves impaired ventricular function (IVF), attributed to various factors such as valvular insufficiency, residual aortic obstruction, arrhythmias, coronary insufficiency, and aortopulmonary collateral burden, presenting clinically with symptoms like failure to thrive, limited exercise tolerance, and arrhythmias. Conversely, the other end entails impairment of Fontan circulation while preserving ventricular function, linked to causes like anatomic obstructions, thromboembolism, elevated pulmonary vascular resistance, pulmonary venous obstruction, and pulmonary arteriovenous malformations, manifesting clinically as pathologic fluid shifts such as endoluminal protein loss (PLE) and plastic bronchitis, as well as cyanosis. Consequently, Fontan physiology may contribute to liver dysfunction and cirrhosis, esophageal varices, renal dysfunction, nutritional derangement, and thromboembolic events [4].

Aside from Fontan circulation impaired by chronic heart failure, Fontan patients experience specific co-morbidities such as plastic bronchitis, intractable arrhythmias, and thrombosis. Also, in the Fontan patient who develops progressive heart failure more than a decade after the Fontan procedure, the potential for hepatic fibrosis becomes an increasing concern as an important risk factor for post-transplant mortality [7,8].

Fontan circulation is likely to exert a pervasive and relentless impact on all organ systems, potentially leading to a gradual deterioration in functionality across multiple domains [9]. Nevertheless, a better characterization of the end-organ consequences of Fontan circulation is necessary and management strategies are emerging in order to reduce or eliminate the development of these potentially life-threatening challenges [10].

Regrettably, previous studies showed that heart transplantation in congenital heart disease had poorer results compared to non-congenital patients, with Fontan circulation being one of the main risk factors for early mortality [11,12,13,14,15].

Additionally, Fontan patients are often human-leukocyte-antigen-sensitized, which is known to be associated with a decreased 1-year post-transplant survival [16,17]. Furthermore, the challenges associated with re-operative cardiac surgery and potential requirement for reconstruction of the great arteries may further complicate their management.

Hence, Fontan circulation is perceived as a higher risk factor in cardiac transplantation compared to other bi-ventricular congenital heart diseases. The purpose of this study is to evaluate our very-long-term results of heart transplantation (HTX) in patients who have been previously palliated with the Fontan procedure. Their survival curve after heart transplantation was compared with that of patients who underwent the same treatment (heart transplantation) without prior palliation with Fontan circulation.

## 2. Materials and Methods

We conducted a retrospective, observational and single-center study, analyzing the results of patients who underwent heart transplantation with underlying congenital heart diseases between 1987 and 2018 at the Papa Giovanni XXIII Hospital in Bergamo, Italy. The patients were divided into two groups according to their circulation pathway at the time of HTX: the Failing Fontan Group (FFG) and the Biventricular Congenital Group (BCG).

To minimize the impact of external factors on the results, patients who had mechanical circulatory assistance before the HTX were excluded. The rationale behind this exclusion stems from the lack of standardized protocols regarding the utilization of such devices until 2018, the year of the last patient recruitment for this study. Consequently, their usage was characterized by variability and lacked uniform guidelines, leading to heterogeneous practices.

All data including demographical characteristics, primary anatomical diagnosis, indication for HTX and post-operative results were collected in an Institutional Database and rigorously evaluated. Data were gathered from patients’ records or by telephone interviews. This study has been approved by the Ethics Committee (EC) of Comitato Etico Territoriale Lombardia 6 (0055233/23 Prot HTXFONTAN, 31 October 2023) in compliance with ethical standards and guidelines.

### Statistical Analysis

Distribution normality was analysed with the Kolmogorov–Smirnov test. Continuous variables were compared using independent Student’s *t*-test with a two-tailed distribution if normally distributed. The Mann–Whitney U-test was used for not normally distributed variables. Categorical variables were compared using chi-square χ^2^ or Fisher’s exact test as needed. The survival differences between the two groups were depicted and compared using the Kaplan–Meier method and log-rank tests. The univariable Cox proportional hazard regression models were used to investigate the effect of pre-operative and post-operative variables on all-cause mortality in the entire cohort of patients. A *p*-value ≤ 0.05 was considered statistically significant. Excel Microsoft 365 MSO (Version 2306 (Build 16529.20226), August 2023 (Microsoft, Redmond, WA, USA) was used for data extraction, and all analyses were performed in R, version 4.3.1 (R Software for Statistical Computing, Vienna, Austria) within RStudio. The R packages used were “survival”, “survminer”, “dplyr” and “ggplot2”.

## 3. Results

The entire cohort included 66 patients who underwent heart transplantation (HTX) for congenital heart disease during the study period. They were divided into two groups according to their pre-heart transplantation circulation physiology: the Failing Fontan Group (FFG), who were diagnosed with univentricular congenital heart disease and had undergone palliative surgery with the Fontan procedure; and the Biventricular Congenital Group (BCG).

The FFG included 29 patients, 16 of whom were male (62%). The mean age at Fontan completion was 6.7 ± 4.8 years old and the mean age at heart transplantation was 17.6 ± 7.9 years. The type of Fontan palliation was the classic atrio-pulmonary connection for 4 patients (13%), the intra-cardiac tunnel in 11 (37%), and the extracardiac connection in 14 (48%). The primary anatomical diagnoses for FFG are depicted in Table 1.

**Figure 1:**
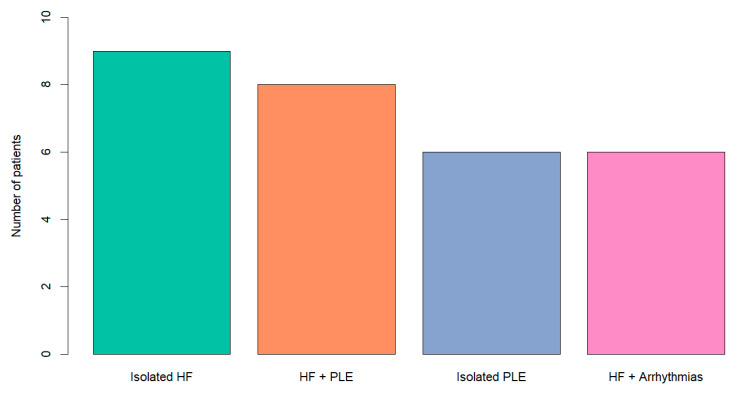
Indication for heart transplantation for Failing Fontan Group. HF: heart failure; PLE: protein losing enteropathy.

The clinical manifestations of Fontan Failure patients and thus indications for HTX in this group were isolated heart failure (HF) in nine patients (31%), HF combined with Protein Losing Enteropathy (PLE) in eight patients (27%), isolated PLE in six patients (20%) and HF with arrythmias in six patients (20%), as shown in Figure 1.

The BCG group comprised 37 patients including 22 males (60%) and the mean age at heart transplantation was 22.5 ± 15.2 years. For those patients, the HTX indication was HF not amenable to conventional HF therapy. The primary anatomical diagnoses for BCG are depicted in Table 2.

Deaths before 30 days were 7/66 (10.6%). There were four (13.8%) early mortalities among the FFG and three (8.1%) in the BCG (*p* value: 0.69).

The causes of early death after HTX in the FFG were cardiogenic shock in two patients, uncontrolled hemorrhage in one patient, and respiratory failure in one patient.

**Figure 2:**
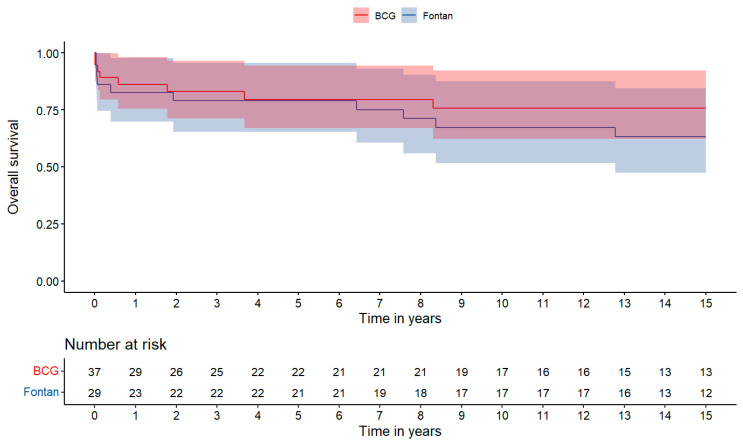
Kaplan–Meier survival curve following cardiac transplantation. Comparison between Failing Fontan Group (FFG) and Biventricular Congenital Group (BCG). Numbers at risk shown above x-axis.

Overall survival was 82.6 ± 13.9% at 1 year, 79.0 ± 14.9% at 5 years, 67.2 ± 17.6% at 10 years and 63.2 ± 18.2 at 15 years for the FFG, and 86.1 ± 11.4% at 1 year, 79.5 ± 13.7% at 5 years, 75.7 ± 14.9% at 10 years, 75.7 ± 14.9% at 15 years for the BCG, with no statistically significant difference (Mantel Cox *p* values: 0.69, 0.89, 0.52 and 0.39, respectively; Figure 2).

Deaths after one year in FFG were due to chronic rejection in two patients, neoplasia in one patient, and cardiac allograft vasculopathy in two patients.

In a sub-analysis of the FCG, the 30-day, 1-year and 5-year survival rates did not change significantly regarding the HTX period, as shown in Table 3.

Table 4 depicts mortality at different timepoints among Failing Fontan Group patients based on the pre-HTX Fontan conduit.

**Figure 3:**
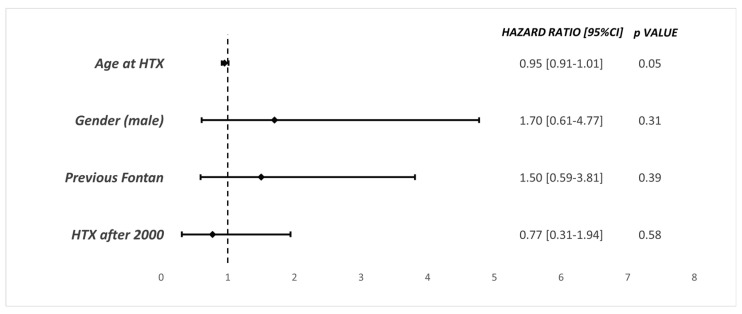
Cox-regression analysis for long-term survival of entire cohort. HR: hazard ratio; CI: confident interval.

Figure 3 shows Cox-regression analysis’s findings of independent predictors of mortality at last follow-up. Although a pre-existing Fontan circulation was a hazard, it was not statistically significant (HR: 1.50, 95%CI 0.59–3.81, *p*: 0.39). There were no differences in terms of long-term outcomes in patients who underwent heart transplantation before or after the year 2000 (HR 0.77, 95%CI 0.31–1.94, *p* value: 0.58). In addition, some generic variables such as the age at the time of transplantation and the patient’s gender did not reach statistical significance.

## 4. Discussion

Considering all physiological and pathological aspects outlined in the Introduction, it can be inferred that while the Fontan procedure enhances survival among patients afflicted with univentricular heart disease, it remains primarily palliative in nature and it should be considered a transitional step to the final treatment, which would be heart transplantation. Nonetheless, as increasing numbers of patients with complex single-ventricle physiology are palliated with the Fontan procedure, the number of children and young adults requiring HTX is expected to increase [5].

The perception of Fontan circulation as a post-transplantation risk factor persists, notwithstanding the absence of conclusive scientific evidence, thus constituting an ongoing subject of scholarly debate.

Previous studies have shown a significantly higher risk for Fontan patients who underwent HTX. Michielon et al. found a higher post-operative hemorrhage rate and low long-term survival compared to other HTX candidate [18]. Inversely, other authors have shown more encouraging results: Gamba et al. demonstrated an overall survival of 71% at 5 years in a case series of 14 patients [19].

The aim of our study is to assess the impact of cardiac transplantation on the natural history of the univentricular heart by analyzing, albeit with limited numbers, whether it improves distant outcomes in terms of expected survival. In the literature, as this is a subject that has been scarcely studied in depth due to the limited numbers, we can observe few studies, the vast majority of which are retrospective and not randomized, with an average follow-up of no more than 10 years.

In our study, the overall survival for the same typology of patients after 5 years from HTX was 79 ± 14.9% and 67.2 ± 17.6% at 10 years. These results are consistent, and they a with other studies including a recent meta-analysis involving 18 articles and 691 patients [20], which showed an overall survival after 5 and 10 years of 69% and 61%, respectively. Our 15 years’ follow-up showed a survival rate of 63.2 ± 18.2%. To the best of our knowledge, this is the longest follow-up in the literature including HTX for Fontan failure patients.

To assess the outcomes of this population, we opted to compare it with a distinct cohort (patients with congenital heart diseases not subjected to Fontan palliation) who underwent similar treatment (heart transplantation), aiming to ascertain if the survival curve differed significantly between the two populations.

Comparing the survival estimated curves of the FFG with the BCG, the survival at 1 year was not statistically significant (*p* 0.69). Furthermore, no significant differences were revealed by extending the follow-up to 5, 10 and 15 years. However, the numbers of events were small in both groups; thus, this study was not able to directly make this comparison.

In the Section 3, we described the causes of death in FFG. Those are comparable to other studies: Voeller et al. identified acute rejection, infection, primary graft dysfunction and bleeding as the primary causes of death after HTX in Fontan failure patients in similar ratios as those of our results [21]. With regard to the late complications, the causes of death in the FFG were no different from those in the BCG, indicating that underlying Fontan circulation caused no additional hinderances after HTX in the long term.

There are no studies in the literature which examine in detail hospitalization-free survival related to cardiac transplantation, nor are there any studies which achieve such an extensive follow-up. It is therefore impossible to make a comparison with other studies.

When the era of HTX was examined, that is, transplantation before and after the year 2000, both groups showed a similar mortality rate at 30 days, 1 year and 5 years (Table 3), and via Cox regression analysis (Figure 3, heart transplantation after 2000, hazard ratio 0.77 [95%CI 0.31–1.94]). These results were similar to those in previous studies, with Davies et al. [6] showing a 30-day mortality of 34.8% before 1999, compared to a 10% mortality in patients transplanted after 1999 (*p* value of 0.06). Controversially, in a 2023 retrospective analysis encompassing adult Fontan patients following heart transplantation across 15 hospitals in the United States and Canada, Lewis observed a contentious trend: post-transplantation survival exhibited improvement over time. Notably, there emerged a significant disparity in survival rates across different decades of transplantation, with the year 2000 serving as the delineating threshold for this analysis [22]. The rationale behind this discrepancy is believed to stem from multiple variables: the demographic characteristics of the studied population, divergent protocols regarding listing duration and transplantation prioritization between Europe and America, and, lastly, the discrepant sample sizes under examination.

Arguably, the way to improve the outcomes in this subset of patients is to list them for heart transplantation sufficiently early. One of the landmark signs of Fontan failure is protein-losing enteropathy (PLE). Freedom from PLE at 10, 20 and 30 years after Fontan procedure was 92%, 89% and 83%, respectively. Survival at 10 and 20 years after diagnosis of PLE is very poor, 35% and 19%, suggesting that an aggressive management of PLE and an earlier consideration for heart transplantation should be considered, especially if PLE is refractory to medical management [3]. Interestingly, in our cohort, PLE was diagnosed in 14/29 patients (48.3%, six patients in isolated form and eight patients with heart failure) and no differences in mortality after HTX were noted between patients with underlying PLE and those without. The reversibility of PLE after HTX has been described after heart transplantation [23]. Unfortunately, we do not have data on the time of onset of PLE in our cohort, and therefore it is not possible to speculate on the perceivable benefits of earlier referral for HTX in these patients.

It is imperative to underscore the necessity of establishing regular surveillance and screening protocols for detecting end-organ dysfunction in Fontan patients both before and after their transition to adult care. This involves implementing comprehensive assessments that encompass cardiac, hepatic, renal, and pulmonary functions given the complex nature of Fontan circulation and its potential long-term impacts on various organ systems. By retrospectively analyzing patient data from the Australia and New Zealand Fontan Registry, Wilson and colleagues found that most patients who transitioned from a tertiary pediatric center to an adult center within the last 5 years did not undergo routine surveillance for end-organ dysfunction [24].

Since these patients were primarily under the care of pediatric cardiologists initially, it was crucial to facilitate a smooth transition to adult care involving a multidisciplinary team. This team should include adult congenital cardiologists and various subspecialists, some of whom may not yet be acquainted with the pathophysiology or the systemic effects of Fontan circulation. These specialists include not only cardiac surgeons who are experts in the field of Grown-Up Congenital Heart Disease (GUCH) but also pulmonologists, gastroenterologists, nutritionists, psychologists, and neurologists [25].

A recent multicentric American study has underscored that specialized expertise correlates with enhanced waitlist outcomes for congenital heart disease (CHD) patients awaiting transplantation. Additionally, post-transplant survival rates were found to be higher at the most high-volume regional center. These results imply a potential benefit of regionalizing CHD transplantation services [26].

Heart transplantation in patients with congenital heart defects who have undergone Fontan palliation remains a complex clinical scenario. Without large-scale analyses of adults with single-ventricle CHD undergoing heart transplantation, little evidence exists to guide listing practices and patient counselling. While this study does not aim to categorize all recipients of heart transplantation uniformly, it is imperative to destigmatize specific recipient cohorts, notably individuals who have undergone Fontan procedures.

This process has already been initiated by previous studies. For instance, in the adult population (>18 years), Bakhtiyar demonstrated that 10-year conditional survival rates were comparable for both biventricular and most single-ventricle CHD patients. Substantially, the survival rates were notably superior for biventricular CHD patients compared to non-CHD heart transplant recipients [27].

Also, pediatric Fontan post-transplantation outcomes are promising, although early mortality remains high, as shown by Kulshrestha. In his paper, the author explained that post-transplant mortality occurs most commonly in the first six months and in decompensated patients. Patients without a critical state can expect 1-year survival >90% [28]. Our research corroborates this, as we observed an overall one-year mortality rate of 82.6 ± 13.9%, without differentiation between compensated and decompensated patients.

Future research directions should be focused on the optimization of referral timing in order to balance the risks of early listing against the potential from deteriorating condition and decompensation. Furthermore, ongoing research should investigate the potential advantages of Fontan revision strategies and the assistance provided by support devices, including Extracorporeal Membrane Oxygenation (ECMO) and Left Ventricular Assist Device (LVAD).

Finally, the establishment of a multicenter, and preferably international, registry is imperative to provide a comprehensive understanding of this complex scenario, addressing the limitations posed by inadequate patient recruitment in individual studies and facilitating the development of guidelines and protocols.

## 5. Conclusions

Despite the Failing Fontan Group seemingly having a higher risk of death in the early post-operative period compared to the Biventricular Congenital Group, the medium- and long-term survival rates are comparable between the two groups. Although pre-heart transplantation Fontan circulation is a hazard, it is not statistically significant. The overall outcomes of HTX for congenital heart diseases are improving with time and, although there is an initial lag amongst Fontan patients, HTX is definitely a viable option for them. With further persistence and refinement of treatment, including mechanical support while on the waiting list, we should expect even better results than current ones.

## Figures and Tables

**Table 1:** Baseline FFG characteristics. AT = Tricuspid atresia; DILV: Double-inlet left ventricle; HLHS: hypoplastic left heart syndrome; TGA: Transposition of great arteries; AP: pulmonary atresia.

Demographic		Failing Fontan Group (FFG)
Number of patients		29
Gender (male)		18 (62%)
Age at Fontan procedure, years		6.7 ± 7.4
Age at heart transplantation, years		17.6 ± 7.9
Previous sternotomy		29 (100%)
Primary anatomical diagnoses	AT	7
	DILV	5
	HLHS	5
	AT + TGA	4
	AP	2
	Others	6
Single-ventricle physiology at heart transplantation		29 (100%)
Fontan conduit	Classic	4 (14%)
	Intra-cardiac tunnel	11 (38%)
	Extra-cardiac tunnel	14 (48%)
Pre-heart transplantation catheterization	Trans-pulmonary gradient (mmHg), mean	6.42 ± 2.70
	Pulmonary artery pressure (mmHg), mean	13.57 ± 4.24

**Table 2:** Baseline BCG characteristics. AVSD: Atrioventricular septal defect; TGA: Transposition of great arteries; ccTGA: congenital corrected TGA.

Demographic		Biventricular Congenital Group (BCG)
Number of patients		37
Gender (male)		22 (60%)
Age at heart transplantation, years		22.5 ± 15.2
Previous sternotomy		29 (78%)
Primary anatomical diagnoses	ccTGA	8
	TGA	7
	Fallot tetralogy	6
	Ebstein	5
	AVSD	4
	Others	7

**Table 3:** Mortality by era of transplantation among Failing Fontan patients. Pts: patients; n.s. not significant (Fisher’s exact test).

Mortality	Before 2000	After 2000	*p* Value
30-day	1/10 pts	3/19 pts	n.s.
1-year	1/10 pts	4/18 pts	n.s.
5-year	2/10 pts	4/17 pts	n.s.

**Table 4:** Mortality at different timepoint among patients with Failure Fontan patients. Pts: patients.

Variable	30-Day Mortality	1-Year Mortality
Classic	0/4 pts	0/4 pts
Lateral tunnel	1/11 pts	2/11 pts
Extracardiac	3/14 pts	3/13 pts

## Data Availability

The data supporting this article will be shared on reasonable request to the corresponding author.
